# Beneficial effects of adding magnesium to desalinated drinking water on metabolic and insulin resistance parameters among patients with type 2 diabetes mellitus: a randomized controlled clinical trial

**DOI:** 10.1038/s41545-022-00207-9

**Published:** 2022-11-12

**Authors:** Waleed I. Albaker, Mohammed T. Al-Hariri, Abdulmohsen H. Al Elq, Nuhad A. Alomair, Ahmed S. Alamoudi, Nikalay Voutchkov, Seungwon Ihm, Mohammed A. Namazi, Ahmed A. Alsayyah, Fatima A. AlRubaish, Fadwa T. Alohli, Fatma A. Zainuddin, Anwar A. Alobaidi, Fatimah A. Almuzain, Mohamed O. Elamin, Naela B. Alamoudi, Mashael A. Alamer, Abdulrahman A. Alghamdi, Nafie A. AlRubaish

**Affiliations:** 1 Department of Internal Medicine, College of Medicine, Imam Abdulrahman Bin Faisal University, Dammam, Eastern Province Saudi Arabia; 2 Department of Physiology, College of Medicine, Imam Abdulrahman Bin Faisal University, Dammam, Eastern Province Saudi Arabia; 3 Department of Chemistry, Science College, Imam Abdulrahman Bin Faisal University, Dammam, Eastern Province Saudi Arabia; 4 Desalination Technologies Research Institute, Saline Water Conversation Corporation, Riyadh, Saudi Arabia; 5 Department of Pathology, College of Medicine, Imam Abdulrahman Bin Faisal University, Dammam, Eastern Province Saudi Arabia; 6 Department of Family Medicine, Armed Forces Hospital, Dhahran, Eastern Province Saudi Arabia; 7 Department of Medical Allied Services, Imam Abdulrahman Bin Faisal University -King Fahd Hospital of the University Eastern Province, Dammam, Saudi Arabia; 8 Department of Biochemistry, College of Medicine, Imam Abdulrahman Bin Faisal University, Dammam, Eastern Province Saudi Arabia; 9 College of Medicine, Imam Abdulrahman Bin Faisal University, Dammam, Eastern Province Saudi Arabia; 10 Intern, College of Medicine, Imam Abdulrahman Bin Faisal University, Dammam, Eastern Province Saudi Arabia; 11 Present Address: Emergency Medicine Department, King Fahad Specialist Hospital, Eastern Health Cluster, Dammam, Eastern Province Saudi Arabia

**Keywords:** Hydrology, Psychology and behaviour

## Abstract

There is evidence that increasing the consumption of water containing magnesium can improve glucose metabolism and insulin resistance in patients with type 2 diabetes mellitus (T2DM). This trial was undertaken with the objective of evaluating the effect of adding different concentrations of magnesium chloride to the desalinated drinking water on the glycemic, metabolic, and insulin resistance parameters among patients with T2DM. A randomized cross-sectional controlled clinical trial was conducted to evaluate the effects of adding magnesium chloride supplement to desalinated drinking water consumed by patients with T2DM on the glycemic and metabolic parameters and indicators of insulin sensitivity. The total number of patients with T2DM who successfully completed the trial is 102. Patients were randomly allocated into three groups: the first group received bottled water without added magnesium (0 mg/L) (Group A, *n* = 37); the second group received bottled water with a low level of magnesium (20 mg/L) (Group B, *n* = 33); and the third group received drinking water with a high level of magnesium (50 mg/L) (Group C, *n* = 32). The daily consumption of elemental magnesium for a period of 3 months resulted in significant improvement in HbA1C (8.0 vs 8.2%, *p* = 0.04), insulin level (7.5 vs 9.9 μIU/mL, *p* = 0.03), and homeostasis model assessment-estimated insulin resistance (HOMA.IR) (2.5 vs 2.9, *p* = 0.002) in group C. However, there was no significant improvement in fasting blood glucose (FBS) level or lipid profile. The results of this study suggest that oral magnesium supplementation at the given dose of 50 mg/L daily added to drinking water could improve long-term glycemic control indicators and reduce insulin resistance in patients with T2DM.

## Introduction

Magnesium is an essential cofactor of several enzymes involved in carbohydrate metabolism. It plays an important role in the regulation of insulin action and glucose homeostasis^1^. Magnesium increases insulin sensitivity by regulating the tyrosine kinase activity of insulin receptors and autophosphorylation on these receptors^2^. Moreover, magnesium prevents the entry of calcium into adipocytes. Reduced magnesium levels intracellularly, result in the accumulation of calcium in adipocytes, followed by increased inflammation and oxidative stress as well as increased insulin resistance^3,4^. Meanwhile, several studies have reported that insulin lower magnesium renal tubular reabsorption and promotes the shift of magnesium from the extracellular to the intracellular compartments, the case which ultimately results in hypomagnesemia in people with hyperinsulinemia or/and poorly controlled diabetes mellitus^5,6^.

Several studies show that Magnesium level influences vitamin D concentration and can boost its intestinal absorption^7^. Metabolism of vitamin D by hepatic 25-hydroxylation and renal 1α-hydroxylation into the active form of 1,25(OH)2D is a magnesium-dependent process^8^.

Recent global reports about magnesium status in diabetic patients are unavailable. According to a previously published report, the worldwide prevalence of hypomagnesemia among patients with type 2 diabetes mellitus (T2DM) ranged between 13.5 and 47.7% compared with 2.5–15% among healthy controls without diabetes^9^. However, results are inconsistent among the studies and, influenced by many factors such as race, gender, and health status, have shown a significant correlation with hypomagnesemia^10–12^.

A study from Saudi Arabia found that 28% of 285 diabetic patients had Hypomagnesemia^13^. All diabetic patients are at risk of having hypomagnesemia related to hyperglycemia. The plasma magnesium level was found to be inversely correlated with fasting blood glucose level and renal excretion rate of magnesium. This indicates that severe hyperglycemia is associated with decreasing the tubular reabsorption of magnesium. In addition, insulin resistance or/and hyperinsulinemia cause a decline in renal magnesium absorption and increased magnesium excretion^14^. The other most relevant cause of hypomagnesemia in diabetic patients is insufficient magnesium intake^15^.

Desalinated water produced by desalinated plants and consumed by the general population has negligible magnesium content. WHO recommended in its 2011 report that “in circumstances where a supply is moving from a source that has significant levels of calcium and magnesium to low-mineral desalinated water, it would be appropriate to consider remineralizing with calcium and magnesium salts”^16^.

This trial was undertaken with the objective of evaluating the effect of adding different concentrations of magnesium chloride to the desalinated drinking water on the glycemic, metabolic, and insulin resistance parameters among patients with T2DM.

## Results

### Subjects

**Fig. 1:**
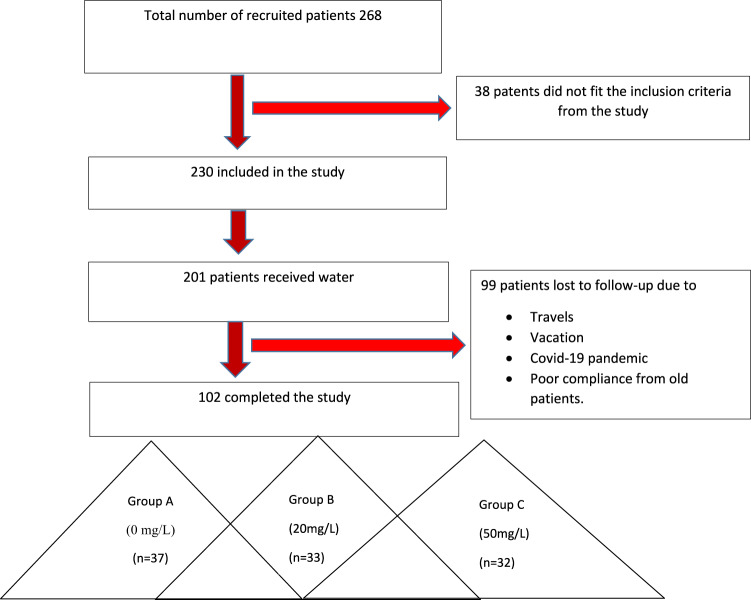
Study flow diagram. The total number of 102 diabetic patients were randomly allocated into Group A (*n* = 37) received bottled water without added magnesium; Group B (*n* = 33) received bottled water with a low level of magnesium (20 mg/L); Group C (*n* = 32) received drinking water with a high level of magnesium (50 mg/L).

Due to the COVID-19 pandemic, the research team overrecruited patients. In total, 268 patients had been recruited (133 male and 135 female). After the baseline visit, 38 patients did not fit the inclusion criteria and were excluded from the study. Therefore, a total of 230 patients (107 male and 123 female) with T2DM diabetes were randomized and included in the study of these 199 patients (95 male and 104 female) have received the magnesium supplement because of failure to allocate the address of the remaining 31 patients (12 male and 19 female) for water bottle delivery. The total number of patients with T2DM who successfully completed the trial is 102 patients (54 males and 48 females), since there were 97 (45 male and 52 female) patient dropouts during the study period. The main reason for the dropout of the 99 patients is the lack of follow-up related to the COVID-19 pandemic. The final distribution of included patients was as follows: 37 patients (15 male and 22 female) were in group A (0 mg/L), which is considered the control group; 33 patients (21 male and 12 female) were in group B (20 mg/L) and 32 patients (18 male and 14 female) in group C (50 mg/L), as shown in Fig. 1.

Post hoc power calculations using a two-tailed paired t-test at an alpha level of 0.05 revealed that even with a sample size of 31 in each group, a power of 0.80 was available to detect a 5% difference in FBG as one of the primary outcome measures.

The magnesium supplement was well tolerated, and there were no side effects due to the supplemented desalinated water bottles apart from one patient from group C who had self-limited diarrhea. Adherence to the supplied water bottle by the patient who completed the study was achieved for all, as assessed by the phone call by using contact details provided on the hospital database.

### Demographic data

Demographic data were analyzed for the 102 patients who completed the study and had the required data performed before and after the study. The median age of all patients was 58 (52, 63) years. There are 37 patients in group A with a mean age of 60 (52, 60) years, 33 patients in group B with a mean age of 59 (54, 63) years, and 32 patients in group C with a mean age of 58 (50, 64) years with no significant statistical difference between the three groups (*p* = 0.283). There is no significant difference in the sex distribution between the three groups *(p* = 0.139). The majority of patients had diabetes for more than 5 years, with more than one-third of the patients (38%) having T2DM for more than 20 years. There is no statistical difference between the three groups in terms of diabetes duration (*p* = 0.826). The percentage of patients with an educational level of high school and college is 43%, again with no statistically significant difference between the groups (*p* = 0.052). The number of patients using insulin with or without oral anti-diabetic agents in the control group is higher than the other groups (62.2%); however, the difference between the three groups is statistically not significant (*p* = 0.800). There is no statistically significant difference between the three groups in any of the clinical and laboratory parameters. The baseline magnesium level is normal in the three groups, with a median magnesium level of 1.81 mg/dl (1.69–1.95) in group A, 1.87 mg/dl (1.72–1.97) in group B, and 1.9 mg/dl (1.7–2.01) in group C (normal range at KFHU of magnesium is 1.6–2.6 mg/dl), which indicates that all patients in this study are not magnesium deficient. Also, the baseline mean of fasting blood glucose (FBG) and baseline mean of glycosylated hemoglobin (HbA1c) in the three groups are high, indicating that patients are having uncontrolled glycemic parameters at the time of entry to the study. All baseline characteristics are presented in Table 1.

**Table 1:** Baseline characteristics of the participants who successfully completed the study.

Characteristics		Control- Group A (0 mg/L) (*n* = 37)	Group B (20 mg/L) (*n* = 33)	Group C (50 mg/L) (*n* = 32)	*p* value
Age (Mean ± SD)		59.20 ± 8.7	57.5 ± 7.04	55.9 ± 8.9	0.283
Sex *n* (%)	Male	15 (40.5)	21 (63.6)	18 (56.2)	0.139
	Female	22 (59.5)	12 (36.4)	14 (43.8)	
Duration of DM n (%)	<1 year	2 (5.4)	2 (6.1)	2 (6.3)	0.826
	1–5 year	3 (8.1)	3 (9.1)	2 (6.3)	
	6–10 year	7 (18.9)	4 (12.1)	9 (28.1)	
	11–15 year	3 (8.1)	6 (18.1)	4 (12.5)	
	16–20 year	8 (21.6)	3 (9.1)	5 (15.6)	
	>20 year	14 (37.8)	15 (45.5)	10 (31.2)	
Education level n (%)	Illiterate	5 (13.5)	1 (3.0)	4 (12.9)	0.052
	High school	14 (37.8)	9 (27.3)	5 (16.1)	
	Less than high school	6 (16.3)	10 (30.3)	3 (9.7)	
	College	12 (32.4)	13 (39.4)	19 (61.3)	
Type of treatment n (%)	Oral	14 (37.8)	14 (42.4)	16 (50.0)	0.800
	Oral and Insulin	19 (51.4)	14 (42.4)	12 (35.5)	
	Insulin	4 (10,8)	5 (15.2)	4 (12.5)	
Clinical and laboratory parameters Median (Interquartile range)	FBG (mg/dL)	145 (115, 179)	146 (128.5, 187)	143 (118, 180)	0.811
	HbA1c (%)	8.2 (7.1, 9.8)	8.3 (7.3, 9.3)	8.2 (7.5, 8.9)	0.662
	Insulin (μIU/mL)	9.0 (5.4, 18.3)	9.1 (5.8, 16.4)	9.9 (5.4, 14.0)	0.911
	C-Peptide (ng/mL)	1.79 (1.30, 2.71)	1.93 (1.20, 2.40)	1.94 (1.48, 3.10)	0.841
	HOMA-IR	2.44 (1.72, 6.60)	3.55 (2.09, 6.50)	2.85 (2.18, 6.90)	0.320
	SBP (mm/Hg)	134 (117, 159)	130.5 (108, 147)	128 (115, 136)	0.488
	DBP (mm/Hg)	78 (72, 85)	75 (70, 87)	75 (73, 81)	0.917
	BMI (Kg/M^2^)	32.7 (27.6, 38.5)	31.39 (29.4, 34.8)	31.3 (28.5, 35.0)	0.814
	TC (mg/dL)	170 (145–199)	167 (150, 180)	159 (127, 180)	0.523
	TG (mg/dL)	117 (84, 155)	116 (94, 177)	111 (83, 144.5)	0.684
	HDL (mg/dL)	45 (36, 52)	48 (40, 54)	40 (35, 49)	0.296
	LDL (mg/dL)	105 (79, 132)	106 (80, 125)	97.5 (72.5, 130.5)	0.884
	Mg (mg/dL)	1.81 (1.69, 1.95)	1.87 (1.72, 1.97)	1.9 (1.7, 2.01)	0.584
	Ca (mg/dL)	9.2 (9.0, 9.4)	9.3 (9.1, 9.7)	9.2 (9.0, 9.5)	0.932
	Vitamin D	25.8 (15.6, 30.1)	23.7 (16.7, 31.9)	29.0 (18.3, 37.9)	0.280

*FBG* fasting blood glucose, *HbA1c* glycosylated hemoglobin, *HOMA-IR* homeostasis model assessment-estimated insulin resistance, *SBP* systolic blood pressure, *DSP* diastolic blood pressure, *BMI* body mass index, *TC* total cholesterol, *TG* triglycerides, *HDL* high-density lipoprotein, *LDL* low-density lipoprotein, *Mg* magnesium, *Ca* calcium.

### Effect on glycemic and insulin sensitivity parameters

Table 2 shows the effect of added magnesium chloride to desalinated water on the glycemic and insulin sensitivity parameters in each group at baseline and after three months of intervention. There is significant improvement in the median level of HbA1c (8.0 vs 8.2%, *p* = 0.04), median insulin levels (7.5 vs 9.9 μIU/mL, *p* = 0.03), and homeostasis model assessment-estimated insulin resistance (HOMA.IR) (2.5 vs 2.9, *p* = 0.002) in group C (high dose) after three months when compared with the baseline value, however, there was no significant changes in FBS level. Although the level of C-peptide improved in group C, the change did not reach statistical significance (2.1 vs 1.9 ng/ml, *p* = 0.720) The same profile has been observed in the glycemic and insulin sensitivity parameters in patients who received a lower concentration of magnesium supplemented of the desalinated water (group B), but the effect did not reach significance. Changes in all glycemic and metabolic control among patients in group A (control group) came to be not significant.

**Table 2:** Effect of different doses of magnesium chloride added to the desalinated water on glycemic and insulin sensitivity parameters.

Glycemic and insulin sensetivity parameters	Control- group A (0 mg/L) (*n* = 37) median (interquartile range)			Group B (20 mg/L) (*n* = 33) median (interquartile range)			Group C (50 mg/L) (*n* = 32) median (interquartile range)		
	Pre	Post	*p* value	Pre	Post	*p* value	Pre	Post	*p* value
FBG (mg/dL)	145 (112, 179)	137 (108, 173)	0.877	147 (129, 200)	141 (121, 179)	0.513	142 (119, 180)	144 (111, 165)	0.651
HbA1c (%)	8.2 (7.1, 9.9)	7.9 (6.8, 8.9)	0.595	8.3 (7.3, 9.3)	7.9 (7.0, 9.1)	0.306	8.2 (7.6, 8.9)	8.0 (7.0, 8.7)*	0.040
Insulin (μIU/mL)	9.0 (5.4, 20.1)	8.9 (6.2, 24.5)	0.075	9.2 (5.8, 17.0)	8.7 (5.8-19.8)	0.930	9.9 (5.4, 16.3)	7.5 (5.7, 12.1)*	0.030
C-Peptide (ng/mL)	1.8 (1.2, 2.8)	1.8 (1.1–2.6)	0.945	1.9 (1.2, 2.4)	2.0 (1.3–2.6)	0.481	1.9 (1.4, 3.1)	2.1 (1.5, 2.7)	0.720
HOMA-IR	2.4 (1.7, 7.2)	3.5 (1.7, 9.6)	0.310	3.6 (2.1, 6.9)	2.9 (2.0-7.5)	0.743	2.9 (2.2, 7.4)	2.5 (1.8, 5.2)*	0.002

*FBG* fasting blood glucose, *HbA1c* glycosylated hemoglobin, *HOMA-IR* homeostasis model assessment-estimated insulin resistance.

*Denote significant differences in the Wilcoxon test between the baseline and the three months for each group.

For the evaluation of glycemic and insulin sensitivity parameters changes over time in both the intervention and control groups, Delta change was calculated as the difference between baseline value and after three months of intervention, which was compared statistically. Desalinated water with added magnesium significantly lowers the Delta insulin (−0.7, *p* = 0.015) and Delta HOMA-IR (−0.555, *p* = 0.020) in the group that received the high dose of magnesium chloride supplement (50 mg/L) compared to the control group. Meanwhile, there is no statical significant Delta changes in FBS, HbA1c, and C-peptide in Group C. Delta changes in the glycemic and insulin resistance parameters in the low dose group (group B) and the control group (group A) did not reach statistical significance (Table 3).

**Table 3:** Differences in the Delta changes of glycemic and insulin resistance parameters between the study groups.

Glycemic indicators	Group A (control) (*n* = 37) Median (IQR)	Group B (20 mg/L) (*n* = 33) Median (IQR)	Group C (50 mg/L) (*n* = 32) Median (IQR)	*p* value
Δ FBG	2.0 (−25.5, 19.0)	−8.0 (−27.0, 33.0)	3.0 (−43.0, 15.0)	0.921
Δ HbA1c	−0.30 (−0.85, 0.20)	−0.1 (−0.60, 0.40)	−0.45 (−1.20, 0.05)	0.440
Δ Insulin	1.0 (−1.1, 4.5)	0.0 (−2.5, 2.0)	−0.7 (−4.4, 0.8)	0.015*
Δ C-Peptide	0.02 (−0.62, 0.57)	0.01 (−0.25, 0.55)	0.09 (−0.36, 0.58)	0.737
Δ HOMA-IR	0.37 (−0.75, 1.91)	−0.11 (−0.91, 1.38)	−0.56 (−1.29, 0.07)	0.020*

*FBG* fasting blood glucose, *HbA1c* glycosylated hemoglobin, *HOMA-IR* homeostasis model assessment-estimated insulin resistance.

*Denote significant differences in the Kruskal–Wallis test between the delta differences after 3 months between the study groups. (*p* < 0.05) compared to the control group.

### Effect on clinical and other laboratory parameters

Magnesium chloride added to the desalinated water has no significant effects on the blood pressure or body mass index in both groups that received a water bottle with magnesium supplementation. The effect on the lipid profile is also not significant except for the unexpected finding of a significant increase in the total cholesterol (*p* = 0.047) among patients who received the high-dose magnesium supplements (group C); this appears to be related to the non-significant increase in the low-density lipoprotein. Also, there was a statistically significant increase in calcium level (*p* = 0.032) among patients who received the high dose of magnesium chloride supplement (50 mg/L) compared to the control group. The change in vitamin D is not significant among the three groups. In addition, there is no statistically significant increase in the magnesium level among patients in the three groups during the intervention (Table 4).

**Table 4:** Effect of different doses of magnesium chloride added to the desalinated water on clinical and other laboratory parameters.

Variables	Group A (0 mg/L (*n* = 37) Median (interquartile range)			Group B (20 mg/L) (*n* = 33) Median (interquartile range)			Group C (50 mg/L) (*n* = 32) Median (interquartile range)		
	Pre	Post	*p* value	Pre	Post	*p* value	Pre	Post	*p* value
SBP (mm/Hg)	133 (117, 158)	130 (115, 142)	0.118	125 (108, 147)	128 (112, 142)	0. 257	126 (116, 134)	125 (114, 135)	0.224
DBP mm/Hg)	77 (72, 84)	80 (71, 84)	0.755	75 (70, 87)	75 (71, 82)	0.722	75 (73, 83)	77 (69, 82)	0.099
BMI (Kg/M^2^)	32.7 (27.3, 38.2)	30.5 (25.3, 36.9)	0.252	32.0 (29.4, 34.8)	30.3 (29.1, 34.9)	0.106	31.1(28.4, 34.8)	29.0 (27.1, 34.5)	0.061
TC (mg/dL)	170 (132, 199)	164 (129.5, 195.0)	0.786	166 (150, 180)	165 (146, 186)	0.905	156 (127, 180	162 (143, 210)	0.047
TG (mg/dL)	118 (85, 163)	118 (84, 156)	0.751	123 (94, 170)	101 (80, 159)	0.888	112 (84, 147)	108 (86, 164)	0.883
HDL (mg/dL)	45 (36, 52)	43 (36, 49)	0.313	48 (40, 54)	46 (35, 51)	0.914	41 (35, 50)	42 (37, 48)	0.665
LDL (mg/dL)	104 (77, 132)	98 (76, 124)	0.566	105 (80, 125)	97 (73, 118)	0.673	94 (72, 130)	102 (83, 153)	0.188
Mg (mg/dL)	1.8 (1.7, 2.0)	1.8 (1.8, 2.1)	0.102	1.9 (1.7, 2.0)	2.0 (1.8, 2.1)	0.053	1.9 (1.7, 2.0)	2.0 (1.8, 2.1)	0.221
Ca (mg/dL)	9.2 (9.0, 9.4)	9.4 (9.1, 9.6)	0.028	9.3 (9.0, 9.7)	9.4 (9.0, 9.7)	0.716	9.3 (9.0, 9.3)	9.4 (9.2, 9.6)	0.032
Vitamin D	25.8 (15.6, 30.1)	22.5 (14.7, 33.0)	0.092	23.7 (16.7, 31.9)	24.4 (16.1, 32.4)	0.905	28.0 (19.0, 37.7)	27.2 (15.7, 35.1)	0.375

*SBP* systolic blood pressure, *DSP* diastolic blood pressure, *BMI* body mass index, *TC* total cholesterol, *TG* triglycerides, *HDL* high-density lipoprotein, *LDL* low-density lipoprotein, *Mg* magnesium, *Ca* calcium.

## Discussion

The objective of this study is to investigate the effects of adding magnesium chloride at different concentrations to the desalinated water regularly consumed by the general population on glycemic, metabolic, and insulin resistance parameters among patients with T2DM. We found that HbA1c, fasting insulin, and HOMA-IR of the group that consumed the high magnesium dose (50 mg/L) are significantly lower than those of the placebo group after three months of ingestion, indicating a significant benefit of the oral magnesium supplement added to the desalinated water in improved insulin sensitivity and long-term glycemic control. Utilizing the pre- and post-intervention Delta changes, there is only a statistically significant in insulin sensitivity parameters. There is also no statistically significant difference in FBS, C-peptide, SBP, DBP, BMI, lipid profile, or magnesium level.

The recommended dietary allowances (RDAs) for magnesium is 310–320 mg per day for adult females and 400–420 mg per day for adult males. However, still, the RDAs for magnesium are low among consumers, suggesting that oral magnesium supplementation is often required in order to achieve an adequate cutoff for the RDAs^17^. Several clinical studies documented the beneficial effects of magnesium supplementation in patients with diabetes mellitus^15^. However, most of the beneficial effects were reported in patients with hypomagnesemia^2,18,19^. As reported, the positive findings of magnesium supplement in diabetes mellitus on HbA1c and insulin sensitivity parameters despite patients having magnesium levels within the normal range at the entry of the study and with no significant change at the end of the study can be explained by the fact that most (99%) of magnesium in the body is stored in the cell and serum magnesium constitute only 1% of the total magnesium in the body^20^. Resnick LM et al. found in their study that total serum magnesium levels do not reflect the magnesium status inside the cells and concluded that serum ionized magnesium or intracellular magnesium depletion can be seen with normal serum concentrations^21^.

Depletion of serum ionized and intracellular magnesium has been seen in many people even with normal total serum magnesium levels, which can be attributed to the lack of sensitivity to total magnesium levels15. In fact, as suggested by a panel of researchers, renal magnesium secretion should be measured particularly when serum magnesium level is lower than 0.82 mmol/L, and magnesium renal excretion of 40–80 mg/day should be considered indicative of magnesium deficiency^22^. Similar to the finding in this study, Mooren et al. carried out a clinical trial and reported that supplementation of oral magnesium improves the sensitivity of insulin even in non-diabetic, normomagnesemic overweight subjects, thus indicating the need for early screening and correction of magnesium levels to prevent type 2 diabetes mellitus^19^. Also, Solati et al., in a clinical trial, reported significant improvement of FBG and post-prandial blood glucose despite no change in the serum magnesium level after administration of 300 mg oral magnesium sulfate (MgSo_4_) supplements for 3 months^23^. The lack of increase in the level of serum magnesium in this study can be explained by the relatively low supplemental dose and the short interventional period, as it was documented in a recent randomized controlled trials study regarding dose- and time-response of oral magnesium supplements, revealed that the maximal response of body magnesium to oral supplementation to achieve a steady-state concentration is at a dose ≥300 mg/day for at least 20 weeks^24^. Also, the improvement in glycemic and insulin sensitivity parameters in this study occurred only in the group that utilized the high-dose supplement. Such a dose-response of dietary magnesium supplement was documented in previous studies^25^.

There is evidence that increased consumption of magnesium-containing water can improve glucose metabolism and insulin resistance. Animal studies have shown that dietary magnesium administration (50 mg/mL in drinking water) for 6 weeks decreased blood glucose levels, improved mitochondrial function, and reduced oxidative stress in diabetic mice^26^. Randomized controlled trials in humans with mineral water suggest that water containing relevant amounts of magnesium can exert an additional effect on glycemic parameters and indicators of insulin sensitivity^27^. Similar to findings in our study, Ham and Shon^28^ showed in an RCT that consumption of natural magnesium-enriched deep sea water containing 350 mg of magnesium per day improves insulin resistance of patients with pre-diabetes where fasting insulin and HOMA-IR values of the patients who consumed balanced deep sea water (BDSW) came to be significantly lower than those of the placebo group after eight weeks. In another study, the effects of magnesium supplementation through water from a salt lake with a high natural magnesium content (7.1% - MAG21) was used after dilution with distilled water to 100 mg/100 mL, 300 mL/day was given to nine mild type 2 diabetic patients with stable glycemic control for 30 days. The supplement resulted in a significant decrease in fasting serum insulin level and HOMA square R^29^.

Improvement of insulin sensitivity parameters among patients with T2DM had been documented in several other studies utilizing oral magnesium supplement^18,30^. The improvement of insulin sensitivity with oral magnesium supplements had been reported even in non-diabetic subjects with insulin resistance^19^.

Despite the use of three 24-h dietary recalls in most of the studies measuring magnesium intake^31^, there is still the potential to be subjected to reporting bias, especially as metabolic syndrome patients have been shown to systematically underreport their food intake^32^. There is also difficulty in adhering to the recommended diet is a common problem in diabetic patients^33^. Therefore, the expected incomplete dietary information of the patients with T2DM in this study may have resulted in our inability to detect clear statistically significant differences in FBG, despite statistically significant improvement in HbA1c among patients who received the high dose of magnesium supplement. Unlike the finding in this study, other studies of oral magnesium supplements demonstrated significant improvement in fasting blood glucose^18,30^. On the other hand, Johnsen SP et al. showed no significant change in glycemic parameters, including HbA1c and fructosamine, in a double-blinded, placebo-controlled, and randomized crossover study of oral magnesium supplement at a dose of 360 mg Mg/day^34^. Also, there was no significant improvement in glucose levels in other studies^35,36^.

In this study, there is a significant improvement of HbA1C without significant improvement in FBG, while Solati et al. reported significant improvement in FBG and post-prandial blood glucose without significant changes in HbA1C^23^. Such a finding is most likely related to the differences in the methodology of the two studies. Systematic review and meta-analysis of randomized controlled trials on the effects of magnesium supplementation on insulin sensitivity and glucose control reported from subgroup analysis that magnesium supplementation for ≥4 months significantly improves the HOMA-IR index and fasting glucose, in both diabetic and non-diabetic subjects^37^.

The C-peptide level is indicative of pancreatic reserve and pancreatic function. Similar to the finding in the study conducted by Ham and Shon^28^, there is no significant change in the C-peptide level in their study. However, another study showed significant improvement in C-peptide levels after 3 months of 250 mg/day of elemental magnesium supplement^30^. Again, the inconsistency in the reported findings related to the change in C-peptide can be related to the differences in the dose and duration of magnesium supplements in addition to the difference in baseline characteristics of patients included in those studies.

According to the United States Food and Drug Administration (FDA), the claims regarding the consumption of magnesium and a reduced risk of hypertension is inconsistent^38^. Meanwhile, several recent studies have shown inverse associations of increased magnesium with developing hypertension^12,39,40^.

In this study, there is no significant change in systolic nor diastolic blood pressure in the three studied groups. A previous qualitative overview of the observational studies points to a negative association between dietary magnesium intake and BP^41^. A recent systematic review and meta-analysis of randomized controlled trials of oral magnesium supplements showed a significant reduction of systolic and diastolic blood pressure. However, subgroup analysis by the dose of intervention, intervention duration, and type of intervention suggested that magnesium supplementation for more than 12 weeks, in doses higher than 300 mg/day or inorganic forms, could significantly decrease both systolic and diastolic blood pressure in T2DM patients^42^.

Purvis JR et al. found in a 16-week randomized, double-blind, placebo-controlled crossover trial on 28 patients with T2DM utilizing sustained-release magnesium chloride at a dose of 384 mg/d, versus placebo, found a significant reduction in systolic blood pressure but not diastolic blood pressure^35^. On the other hand, daily administration of 300 mg of MgSo4 for 3 months to 54 patients with T2DM significantly improved both systolic and diastolic blood pressure in a double-blinded RCT^23^. Patients with hypertension showed a significant reduction of systolic (*p* < 0.01), diastolic (*p* = 0.0038), and mean (*p* < 0.01) blood pressure after administration of magnesium supplementation through water from a salt lake with a high natural magnesium content (7.1% - MAG21) at a dose of 300 mL/day for 30 days^29^. This suggests that the dose, duration, and type of elemental calcium can influence the effect of oral magnesium supplements on blood pressure among patients with T2DM. According to reported evidences, which attributed morbidity and mortality in cardiovascular diseases to low magnesium intake secondary to drinking desalinated water^43,44^. Recently, FDA has recommended the consumption of magnesium to reduce the risk of hypertension^38^.

A large population-based study from Canada with a nationally representative sample reported a negative association between serum magnesium level and BMI^45^. In this clinical trial, we did not find any significant changes in the BMI of patients who received magnesium chloride. Several other studies reported non-significant changes in body weight or BMI among patients with T2DM after oral magnesium supplements. Several other studies reported non-significant changes in body weight or BMI among patients with T2DM after oral magnesium supplements^18,41^. There is a possibility that a longer duration of magnesium supplement may be required to induce a significant effect on BMI.

Findings related to the change in lipid profile after oral magnesium supplementation to patients with T2DM is also inconsistent. In this study, there is no significant change in the lipid profile among the three studied groups apart from an unexplained finding of a significant increase in the total cholesterol among patients who received the high-dose magnesium supplements (group C), which appears to be related to the non-significant increase in the LDL. In fact, Solati et al.^23^, documented a significant decrease in LDL and non-HDL cholesterol among patients who received magnesium oral supplement compared to patients who received the placebo, while there was no significant changes in other lipid profile. On the other hand, a marked decrease of the mean triglyceride level after supplementation with salt lake water having high natural magnesium content has been reported^29^. Also, total cholesterol and LDL significantly decreased in the BDSW group after eight weeks of BDSW ingestion compared with the placebo group, as reported by Ham and Shon^28^. Findings from the last two studies may point towards the possibility that the favorable effect on lipid profile is more pronounced when the magnesium supplement comes from natural water sources. Other studies reported no significant changes in all parameters of lipids after oral magnesium supplementation^35,36^.

A number of previous studies have evaluated the associations between magnesium supplements and vitamin D levels^46,47^. However, there was no consistent evidence that supplementing with magnesium improved vitamin D metabolite levels^7^. Our data did not detect any significant changes in the vitamin D levels between pre- and post-intervention data. However, this observation can be attributed either to the fact that the dose of magnesium used was small to show significant changes or, the study patients had insufficient vitamin D levels (21–29 ng/mL)^48^. Therefore, the level of vitamin D didn’t substantially increase during short-term magnesium intake because pre-vitamin D3 was not available in sufficient amounts^46^.

Another finding in this study is the significant increase in the serum calcium level despite non-significant changes in the serum magnesium and vitamin D levels. Other studies reported non-significant changes in the serum calcium level^18,23^. Some studies reported no change in calcium levels after using the calcium-to-magnesium ratio^18,30^.

The main strength of this study is the fact that it is the first study in the region evaluating the effect of adding magnesium supplementation to drinking desalinated water. The other strength is the inclusion of a relatively high number of patients in comparison to many studies that evaluated the effect of oral magnesium supplements in patients with T2DM. In addition, this study evaluated the effect of different doses of magnesium supplements. This study is not without limitations, that includes being a single-center study with a high dropout of patients, which was mainly related to the loss of follow-up related to the COVID-19 pandemic. Also, only serum and not intracellular magnesium was measured, which may not be representative of magnesium status or intracellular pool. The magnesium doses used are small, which may result in the lack of a significant favorable effects on other metabolic and biochemical parameters, as documented by dose and time-response studies. Finally, it was not possible to calculate the daily magnesium consumption from other sources.

In conclusion, this clinical trial showed statistically significant improvement of HbA1C as a parameter of long-term glycemic control but not in the short-term control as reflected by the non-significant change in FBS. There is also a significant improvement in insulin resistance, at the given oral magnesium dose of 50 mg/L daily added to drinking water, which also can, in turn, help in glycemic control. This can result in decreased long-term diabetic complications. Based on the findings from this study, there is an initial indication that a magnesium supplement needs to be added to the desalinated drinking water; however, double-blinded MCT utilizing higher doses of magnesium and with longer duration is needed to support such a recommendation.

## Methods

### Study design

This randomized, multi-arm parallel group (1:1:1) controlled clinical trial was conducted by healthcare providers and medical interns to evaluate the effects of adding a magnesium supplement to the desalinated drinking water utilized by patients with T2DM on the glycemic parameters, metabolic parameters, and indicators of insulin sensitivity. Randomization of the patients in each arm of the study was carried out utilizing randomization tables. The study was carried out at King Fahd Hospital of the University (KFHU) AL Khobar, Eastern province, Kingdome of Saudi Arabia (KSA), in collaboration with Saline Water Conversion Corporation Research Institute (SWCCRI), Al-Jubail, KSA, during the period of September 2020 to August 2021. Patients with T2DM who were available and willing to participate during the three months recruitment period were recruited from the Diabetic Clinics of KFHU. The study was approved by the local institutional review boards of Imam Abdulrahman Bin Faisal University, Saudi Arabia (IRB-2019-01-407) and was registered in ClinicalTrials.gov (NCT04632277). All participants heard and understood the full details of the study and voluntarily decided to participate. Informed consent was taken from each subject enrolled in this study, which was performed according to the guidelines of good clinical practice and the declaration of Helsinki.

### Participants

All patients with T2DM on any anti-diabetic therapy aged 18-70 years old, were eligible for the study. The study has excluded patients with type 1 diabetic mellitus, pregnant or lactating women, patients who have malignancy, those using immunosuppressive or corticosteroid therapy, and patients who have renal or hepatic dysfunction. Patients with chronic diarrhea, malabsorption, or with a previous history of major intestinal surgery were also excluded. The sample size was calculated by keeping in mind the mean fasting blood glucose (FBG) in cases and control.

### Preparation of the product and study procedure

**Fig. 2:**
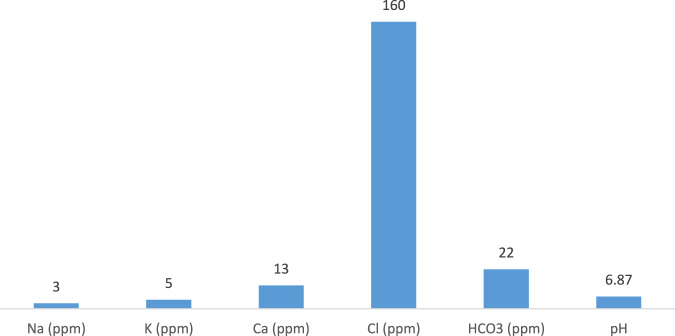
Intervention (bottled water) compositions. Composition of the fixed ingredients in each intervention (bottled water).

Magnesium chloride added to desalinated water bottles had been prepared by SWCCRI, Al-Jubail, and KSA for the purpose of the study. It was prepared in similar bottles but with different concentrations of magnesium content and marked as A, “water without added magnesium (0 mg/L)” which is used as control, B, “Low magnesium bottled water (20 mg/L)”, and C “high magnesium bottled water (50 mg/L)”. The other compositions of intervention (bottled water) are shown in Fig. 2. These doses of magnesium have been chosen after testing different concentrations and making sure that the highest dose will not affect the taste of the water, and it will be palatable when utilized by the study subjects to assure complaints. We used the magnesium chloride solution because magnesium chloride solution shows a higher bioavailability than other commercial magnesium preparations^49^. The volume of each water bottle is one liter. All eligible subjects participated in this study were randomly allotted into one of three groups utilizing computer-generated random numbers and received water bottles with different magnesium concentrations. Patients were blinded to the type of water supplied, and the water bottles were delivered to the homes of patients by a third party who was also blinded to the concentration of magnesium in the water bottles.

All patients were asked to consume one liter of the supplied water/day. When preparing coffee and tea, ordinary drinking water could be used. The intervention lasted for 3 months and adherence to consumption of the supplied water bottles is evaluated through biweekly follow-up phone calls. Patients were also asked about any side effects during the phone calls. None of the participants changed their normal dietary habits and maintained an otherwise normal lifestyle during the trial. Change in the anti-diabetic medications during the intervention period was left to the decision of the treating physician, and it was avoided unless necessary. Dietary evaluation of daily magnesium intake was tried by the clinical dietitian through 3 days of dietary recall, but unfortunately, the response rate was low and the collected data was not representative.

### Data collection

Before their inclusion in the study, all patients were clinically evaluated and laboratory tested in order to determine the presence of any of the exclusion criteria. All participants were seated in an air-conditioned room and had 10–15 min of rest before measurements were taken. After signing the consent form, the socio-demographic data and clinical data were recorded and included: patient address, educational level, smoking history, age, sex, type and duration of diabetes mellitus, and type of anti-diabetic medications. Height and weight were taken using standard protocols with the subjects in light clothing and without shoes. Body mass index (BMI) was calculated as weight (in kilograms) divided by height (in meters squared). The average of three consecutive blood pressure readings with intervals of 5-min rest were obtained, using an automated sphygmomanometer (Dinamap^®^; GE Medical Systems, Milwaukee, WI, USA). All measurements were taken at the baseline and after three months of intervention.

### Blood samples

After 12 h of fasting, blood samples were taken to determine the serum concentration of FBS, glycosylated hemoglobin (HbA1c), fasting C-peptide levels, fasting insulin levels (for patients not on insulin therapy), magnesium, calcium, 25 (OH) vitamin D, and lipid profiled. The HOMA-IR was calculated according to the following formulas: HOMA-IR = (glucose mg × insulin level)/405. Blood samples were collected before and after 3 months of consumption of the desalinated drinking water bottle supplemented with magnesium chloride. Moreover, the Delta changes were calculated as the difference between the maximal and the baseline.

### Statistical analysis

All statistical analyses were performed using the Statistical Package for the Social Sciences (IBM, Chicago, IL, USA), version 23.0. All data were tested by Shapiro–Wilks test for normality assumptions. Median (interquartile range) was calculated for skewed distribution and mean (±standard deviation) was calculated for normally distributed data, while frequencies and percentages were calculated for all categorical data. Wilcoxson and Kruskal–Wallis tests were used to compare skewed data between the pre- and post-intervention data. While ANOVA was used to compare the mean values between groups for normally distributed data. *P* values < 0.05 were considered to be statistically significant.

### Reporting summary

Further information on research design is available in the Nature Research Reporting Summary linked to this article.

## Supplementary information

Reporting Summary

## Data Availability

The data that support the findings of this study are available from the corresponding author upon reasonable request.
