# Supplementary material for: Beneficial effects of adding magnesium to desalinated drinking water on metabolic and insulin resistance parameters among patients with type 2 diabetes mellitus: a randomized controlled clinical trial
Source: NPJ Clean Water. 2022 Nov 12;5(1):63. doi: 10.1038/s41545-022-00207-9 (PMC9660218; doi:10.1038/s41545-022-00207-9)
Supplement: Supplementary file 1 — Reporting Summary [file 41545_2022_207_MOESM1_ESM.pdf]

nature portfolio

Corresponding author(s): Mohammed Al-Hariri

Last updated by author(s): Oct 9, 2022

Reporting Summary

Nature Portfolio wishes to improve the reproducibility of the work that we publish. This form provides structure for consistency and transparency in reporting. For further information on Nature Portfolio policies, see our Editorial Policies and the Editorial Policy Checklist.

Statistics

For all statistical analyses, confirm that the following items are present in the figure legend, table legend, main text, or Methods section.

n/a	Confirmed	
☐	☒	The exact sample size (*n*) for each experimental group/condition, given as a discrete number and unit of measurement
☐	☒	A statement on whether measurements were taken from distinct samples or whether the same sample was measured repeatedly
☐	☒	The statistical test(s) used AND whether they are one- or two-sided<br>*Only common tests should be described solely by name; describe more complex techniques in the Methods section.*
☐	☒	A description of all covariates tested
☐	☒	A description of any assumptions or corrections, such as tests of normality and adjustment for multiple comparisons
☐	☒	A full description of the statistical parameters including central tendency (e.g. means) or other basic estimates (e.g. regression coefficient) AND variation (e.g. standard deviation) or associated estimates of uncertainty (e.g. confidence intervals)
☐	☒	For null hypothesis testing, the test statistic (e.g. *F*, *t*, *r*) with confidence intervals, effect sizes, degrees of freedom and *P* value noted<br>*Give P values as exact values whenever suitable.*
☒	☐	For Bayesian analysis, information on the choice of priors and Markov chain Monte Carlo settings
☒	☐	For hierarchical and complex designs, identification of the appropriate level for tests and full reporting of outcomes
☐	☒	Estimates of effect sizes (e.g. Cohen's *d*, Pearson's *r*), indicating how they were calculated

Our web collection on statistics for biologists contains articles on many of the points above.

Software and code

Policy information about availability of computer code

Data collection	No software was used
Data analysis	All statistical analyses were performed using the Statistical Package for the Social Sciences (IBM, Chicago, IL, USA), version 23.0. All data were tested by Shapiro-Wilks test for normality assumptions. Median (interquartile range) was calculated for skewed distribution, and mean (±standard deviation) was calculated for normal distributed data, while frequencies and percentages were calculated for all categorical data. Wilcoxson and Kruskal-Wallis tests were used to compare skewed data between the pre- and post-intervention data. While ANOVA was used to compare the mean values between groups for normal distributed data. P values <0.05 were considered to be statistically significant.

For manuscripts utilizing custom algorithms or software that are central to the research but not yet described in published literature, software must be made available to editors and reviewers. We strongly encourage code deposition in a community repository (e.g. GitHub). See the Nature Portfolio guidelines for submitting code & software for further information.

Data

Policy information about availability of data

All manuscripts must include a data availability statement. This statement should provide the following information, where applicable:

- Accession codes, unique identifiers, or web links for publicly available datasets
- A description of any restrictions on data availability
- For clinical datasets or third party data, please ensure that the statement adheres to our policy

All data generated or analysed during this study are available from the corresponding author on request.

Field-specific reporting

Please select the one below that is the best fit for your research. If you are not sure, read the appropriate sections before making your selection.

☒ Life sciences ☐ Behavioural & social sciences ☐ Ecological, evolutionary & environmental sciences

For a reference copy of the document with all sections, see nature.com/documents/nr-reporting-summary-flat.pdf

Life sciences study design

All studies must disclose on these points even when the disclosure is negative.

Sample size	Due to the COVID-19 pandemic, the research team over recruited patients. In total, 268 patients had been recruited (133 male and 135 female). However, even with this sample size (n=102), power calculations showed good power (80%) at a 5% significant level to detect differences of any clinically meaningful magnitude in the variables examined in the study.
Data exclusions	The study has excluded patients with type 1 diabetic mellitus, pregnant or lactating women, patients who have malignancy, those using immunosuppressive or corticosteroids therapy, and patients who have renal or hepatic dysfunction. Patients with chronic diarrhea, malabsorption, or with previous history of major intestinal surgery were also excluded
Replication	We confirm that the manuscript has cover all the measurements and preparation of the product as well as study procedure to ensure the reproducibility
Randomization	All eligible subjects participated in this study were randomly allotted into one of three groups utilizing computer-generated random numbers and received water bottles with different magnesium concentrations.
Blinding	Patients were blinded to the type of water supplied, and the water bottles were delivered to the homes of patients by a third party who was also blinded to the concentration of the magnesium in the water bottles.

Reporting for specific materials, systems and methods

We require information from authors about some types of materials, experimental systems and methods used in many studies. Here, indicate whether each material, system or method listed is relevant to your study. If you are not sure if a list item applies to your research, read the appropriate section before selecting a response.

Materials & experimental systems

n/a	Involved in the study	
☒	☐	Antibodies
☒	☐	Eukaryotic cell lines
☒	☐	Palaeontology and archaeology
☒	☐	Animals and other organisms
☐	☒	Human research participants
☐	☒	Clinical data
☒	☐	Dual use research of concern

Methods

n/a	Involved in the study	
☒	☐	ChIP-seq
☒	☐	Flow cytometry
☒	☐	MRI-based neuroimaging

Human research participants

Policy information about studies involving human research participants

Population characteristics	All patients with T2DM on any anti-diabetic therapy aged 18-70 years old, were eligible for the study.
Recruitment	The study was carried out at King Fahd Hospital of the University (KFHU) AL Khobar, Eastern province, Kingdome of Saudi Arabia (KSA) in collaboration with Saline Water Conversion Corporation Research Institute (SWCCRI), Al-Jubail, KSA during the period of September 2020 to August 2021. Patients with T2DM who were available and willing to participate during the three months recruitment period were recruited from the Diabetic Clinics of KFHU.
Ethics oversight	The study was approved by the local institutional review boards of Imam Abdulrahman Bin Faisal University, Saudi Arabia (IRB-2019-01-407) and was registered in the ClinicalTrials.gov (NCT04632277). All participants heard and understood the full details of the study and voluntarily decided to participate. Informed consent was taken from each subject enrolled in this study, which was performed according to the guidelines of good clinical practice and the declaration of Helsinki.

Note that full information on the approval of the study protocol must also be provided in the manuscript.

Clinical data

Policy information about clinical studies

All manuscripts should comply with the ICMJE guidelines for publication of clinical research and a completed CONSORT checklist must be included with all submissions.

Clinical trial registration	ClinicalTrials.gov (NCT04632277)
Study protocol	Magnesium chloride added to desalinated water bottles had been prepared by SWCCRI, Al-Jubail, KSA for the purpose of the study. It was prepared in similar bottles but with different concentration of magnesium content and marked as A "water without added magnesium (0mg/l)" which is used as control, B "Low magnesium bottle water (20mg/l)" and C "high magnesium bottle water (50 mg/l)". The other compositions of intervention (bottled water) are shown in Figure 2. These doses of magnesium have been chosen after testing different concentration and making sure that the highest dose will not affect the taste of the water, and it will be palatable when utilized by the study subjects to assure complaints. We used the magnesium chloride solution because magnesium chloride solution shows a higher bioavailability than other commercial magnesium preparations47. The volume of each water bottle is one liter. All eligible subjects participated in this study were randomly allotted into one of three groups utilizing computer-generated random numbers and received water bottles with different magnesium concentrations. .All patients were asked to consume one liter of the supplied water/day. When preparing coffee and tea, ordinary drinking water could be used. The intervention lasted for 3 months and adherence to consumption of the supplied water bottles is evaluated through biweekly follow-up phone calls. Patients were also asked about any side effects during the phone calls. None of the participants have changed their normal dietary habits and maintained an otherwise normal lifestyle during the trial. Change in the anti-diabetic medications during the intervention period was left to the decision of the treating physician, and it was avoided unless necessary. Dietary evaluation of daily magnesium intake was tried by the clinical dietitian through 3 days dietary recall, but unfortunately, the response rate was low and the collected data was not representative.
Data collection	Before their inclusion at the study, all patients were clinically evaluated and laboratory tested in order to determine the presence of any of the exclusion criteria. All participants were seated in an air-conditioned rooms and had 10-15 min of rest before measurements were taken. After signing the consent form, the socio-demographic data and clinical data were recorded and included: patient address, educational level, smoking history, age, sex, type and duration of diabetes mellitus, and type of anti-diabetic medications. Height and weight were taken using standard protocols with the subjects in light clothing and without shoes. Body mass index (BMI) was calculated as weight (in kilograms) divided by height (in meters squared). The average of three consecutive blood pressure readings with intervals of 5-minute rest were obtained, using an automated sphygmomanometer (Dinamap®; GE Medical systems, Milwaukee, WI, USA). All measurements were taken at the baseline and after three months of intervention.
Outcomes	Fasting Blood Glucose<br>HbA1c<br>Insulin<br>C-Peptide<br>HOMA-IR<br>Ca<br>Vitamin D<br>Lipid profile<br>blood pressure
